# Pro-environmental habits: An underexplored research agenda in sustainability science

**DOI:** 10.1007/s13280-021-01619-6

**Published:** 2021-09-14

**Authors:** Noah Linder, Matteo Giusti, Karl Samuelsson, Stephan Barthel

**Affiliations:** 1 Department of Building Engineering, Energy Systems and Sustainability Science, University of Gävle, Kungsbäcksvägen 47, 801 76 Gävle, Sweden; 2 Stockholm Resilience Centre, Stockholm University, Stockholm, Sweden

**Keywords:** Behaviour change, Climate change, Habit, Pro-environmental behaviour, Urban sustainability

## Abstract

Habits are the fundamental basis for many of our daily actions and can be powerful barriers to behavioural change. Still, habits are not included in most narratives, theories, and interventions applied to sustainable behaviour. One reason societies struggle to reach policy goals and people fail to change towards more pro-environmental lifestyles might be that many behaviours are now bound by strong habits that override knowledge and intentions to act. In this perspective article, we provide three arguments for why pro-environmental habits are a needed research agenda in sustainability science: (1) habit theory highlights how behaviour is heavily reliant on automatic processes, (2) the environmental context sets boundary conditions for behaviour, shape habits, and cues action responses, and (3) our habits and past behaviour shape our values and self-identity. These arguments highlight the transformative potential of looking at sustainable behaviours through a habit lens. We believe a research agenda on pro-environmental habits could generate a more holistic understanding of sustainable behaviours and complement today’s dominating approaches which emphasize reasoned decisions and intrinsic motivations such as values, norms, and intentions to understand and predict pro-environmental behaviour. We highlight evident knowledge gaps and practical benefits of considering habit theory to promote pro-environmental behaviours, and how habit architecture could be utilized as a strong leverage point when designing, modifying, and building urban environments.

## Introduction

Human behaviour is at the root of most environmental challenges we face today. To reach sustainability targets and ensure a safe operating space for humanity on earth, rapid behaviour changes are needed across scales ranging from individuals to leaders on all levels of society (Steffen et al. 2015; UN General Assembly 2015). Promisingly, pro-environmental values seem to grow stronger every day (Bouman and Steg 2019; Manfredo et al. 2020) and recent reports indicate that more than 60% of people across the world now acknowledge the climate crisis (Flynn et al. 2021). Yet, a significant shift towards sustainable lifestyles have not been observed (IPCC 2014; Brondizio et al. 2019). One reason many struggle to change might be that several environmentally damaging behaviours are by now bound by habits that can be strong enough to continuously override new knowledge and intentions (Verplanken 2018). Habits are the fundamental basis for many daily actions and can be powerful barriers to change, once habits take shape they persist without much deliberation or re-consideration (Wood and Rünger 2016). It is therefore not only the decisions that we make today that form the wide foundation of our unsustainable behaviours, but the decisions we once took that are now solidified in strong habits and lifestyles.

However, the impact of habits is seldom accounted for in studies on pro-environmental behaviour, which instead often emphasize the role of values, norms, attitudes, intentions, and motivation for pro-environmental behaviour (Schultz and Kaiser 2012; Sörqvist 2016). Correspondingly, most interventions aiming to ignite sustainable transformations are focussed on building intrinsic motivation through rational processes like knowledge building, feedback, and monitoring—which are likely not powerful enough to break habits and create long-term behavioural change (Verplanken 2018). Attitude–behaviour models (e.g. theory of planned behaviour and value belief norm theory) have been the dominating lens through which to study pro-environmental behaviour and they have shown to be pragmatic and effective, explaining around 20–30% of the variance in human behaviour despite inherent vast complexities involved (see e.g. McEachan et al. 2016). However, they seldom paint the full picture and people often fail to align knowledge and internal motivations with sustainable actions (Kollmuss and Agyeman 2002; Steg and Vlek 2009). Such value/attitude–action gaps (Blake 1999) mean that even though an individual possesses intrinsic motivation she will not necessarily manifest such motivational drive in pro-environmental behaviours. Some studies have even shown the opposite to be true. For example, households of higher socio-economic status often report strong pro-environmental values and relatively higher ecological knowledge, while at the same time energy consumption is strongly correlated with house size, a key indicator of socio-economic class (Jackson 2004).

Habits seem to be largely neglected within the field of sustainability science, even though they have been highlighted as a potential barrier for aligning intrinsic motivation with sustainable behaviour changes (Verplanken et al. 1998; Kollmuss and Agyeman 2002; Jackson 2004), and as setting boundary conditions for the validity of attitude–behaviour models (Verplanken and Aarts 1999). A Scopus search undertaken on 26 January 2021 showed that in the vast amount of research addressing sustainability (596 653 articles),^1^ less than 0.5% articulate habits in the title, abstract, or keywords. In the research explicitly addressing pro-environmental behaviour (2719 articles),^2^ only about 3% of the articles address habits. A closer look at the latter search revealed that most of these articles either only briefly mention habit, use habits to simply refer to behaviour or as a measure of repeated past behaviours. Some efforts have been made to expand attitude–behaviour models by including habits, often increasing the predictive power (e.g. Donald et al. 2014; Chuang et al. 2018; Bell and Ulhas 2020; Çoker and van der Linden 2020; Liu et al. 2020; Aboelmaged 2021). Others call for more research on habits (Peattie 2010; Wynes et al. 2018) or highlighting habits as a strong barrier/motivator for pro-environmental behaviours (e.g. Dharmesti et al. 2020; Huang et al. 2020; Russell and Knoeri 2020), and still some others draw on habit theory when designing behaviour-specific interventions (Staats et al. 2004; Winter and Burn 2010; Ro et al. 2017; Heidbreder et al. 2020). We only found a few articles addressing the roles of habits for sustainability transformations. For example, White et al. (2019) include habits in a framework for designing interventions to promote sustainability transformations and Dahlstrand and Biel (1997) highlight different propensity levels for shifts towards sustainable behaviour depending on the strength of habits. Within the scope of this article, we limited the Scopus search to “pro-environmental behaviour”. It is possible, however, that habits are also considered in conjunction with other terms, not captured in this search, such as “green behaviour”, “sustainable behaviour”, or specific behaviours such as “recycling”. Nevertheless, our search indicates a seeming lack of scientific interest in the role of habits in relation to sustainable actions from most scholars. This is surprising considering the powerful influence habit can have on behaviours that must radically change in the near future.

This paper aims to explore the potential habits may have for igniting (or hindering) transformations towards sustainable behaviours. We define Pro-Environmental Habits (PEH) as “habits that either benefit the environment or harms it as little as possible” (based on the Steg and Vlek (2009) definition of pro-environmental behaviour). We start by giving an account of the existing theories and research on habits. We then discuss habits in relation to pro-environmental behaviours and articulate three arguments for why research on PEH may be a needed research agenda for sustainability science. We discuss implications, research gaps, and argue for the potential of focussing more on breaking habits and on building PEH when designing and modifying the built environment.

## Our reliance on habits

In our daily lives, we develop habits through repeating actions in stable contexts, which then become efficient, default modes of responses—persisting with little guidance, intentions, or deliberate thought (Gardner 2015; Wood and Rünger 2016). Habits guide many decisions, with one study looking at hourly self-reported behaviour records estimating that about 40% of all our daily behaviours are guided by habit without conscious deliberation (Wood et al. 2002). This is likely a low estimate considering that self-reported measures struggle to fully capture automatic behaviours. Habits and other automatic processes often remain understudied since they are inherently hard to measure (Rebar et al. 2018) and can require expensive longitudinal analysis (Gardner 2015). It might be hard to estimate exactly how much of our behaviour is governed by habits, but it is safe to say that habits guide many of our daily actions.

Our reliance on habits is not that surprising considering how they free up working memory and enable us to save time and multi-task (Wood and Rünger 2016). Habits can motivate us to act when we are low on willpower, stressed, or not able to deliberate on responses (Mazar and Wood 2018). Habitual knowledge is also stable and protected from short-term whims or random events, and can provide pre-determined action responses or mental solutions to recurring complex problems (Wood and Rünger 2016). Habits can even be seen as our brain’s way of outsourcing action control to environmental cues, which provides us with a ready response to familiar situations (ibid). Reliance on habits is logical from an evolutionarily perspective, as we are evolved to preserve time and energy and the use of cognitive attention and working memory is both energy-draining and time-consuming (Epstein 1994). In addition to habits, we also use e.g. heuristics (mental rules of thumb), norms, and emotions to help guide actions without much need for deliberation (Strack and Deutsch 2004). Such fast, automatic, intuitive, and energy-efficient ways of handling information or acting on stimuli have been conceptualized as being part of a “system 1” or the “impulsive” system. “System 2” or the “reflective” system on the other hand is our slow, deliberate, conscious, and more energy draining system (ibid.).

It is not always the case that relying on our impulsive system is working to our advantage. Problems arise when we, for example, develop unwanted habits that are hard to break, our heuristics systematically arrive at wrong or biassed conclusions or when we respond to our emotions with regretful actions. One reason habits are fundamentally difficult to “un-do” is that they are controlled by different neural networks compared to intention-driven behaviour: after a habit is established the behaviour should no longer be considered goal-directed (Miller et al. 2019) and tends to persist whether we want it to or not. As Nathaniel Emmons put it: *“Habit is either the best of servants or the worst of masters”* (Edwards 1891, p. 212)*.* Only through effortful goal pursuits, for example when the habit proves unreliable in a given context or when people are especially motivated and able to tailor responses to particular circumstances, do we sometimes manage to change. However, seeing how willpower fluctuates over time, motivation alone often fails to change established habits. Various factors such as time pressure, distraction, stress, hunger, and addiction impede people’s ability to deliberate and consciously choose their actions, thus tipping the balance back towards established automatic habits (Verplanken 2018).

### Habit theory, formation, and mechanisms

Habits are commonly used in the literature without a clear definition (Southerton 2013), often used simply as a measure of repeated past behaviour (Verplanken 2018). However, three key pillars are constituting habits: they need repetition to form, they direct behaviour automatically, and they are context-dependent (e.g. Kurz et al. 2015). Hence, habits can be defined as *“memory-based propensities to respond automatically to specific cues, which are acquired by the repetition of cue-specific behaviour in stable contexts”* (Verplanken 2018, p. 4). It is important to note here that habits are also explored from different perspectives in the literature and that there is still some unanswered questions and disagreements about what habits are. For example, social practice scientists would not recognize habits through context-cued automaticity. Instead, they look at habit without separating the individual and the context and see habits as constantly unfolding actions, an integrated part of social processes and complex dynamics (Kurz et al. 2015). In this paper, we take a psychological perspective on habits using the definition from Verplanken (2018) presented above.

The formation of habits is often, at first, motivated by peoples’ deliberate choice to achieve specific goals (Wood and Rünger 2016). For example, an intention to floss can lead to the habit of flossing after teeth brushing (Judah et al. 2013). However, we may also develop many habits from behaviours we do not necessarily intend to do. For example, a lack of available public transportation can push a habit of car commuting regardless of preferences and a tight budget can form dietary habits around cheaper less nutritious food. People even develop habits for maladaptive behaviours that they explicitly intend *not* to do, such as procrastination or nail-biting (Verhoeven and de Wit 2018) and can fall into the habits of *not* doing something (however, these kinds of habits are less explored in the literature; Verplanken 2018).

**Fig. 1:**
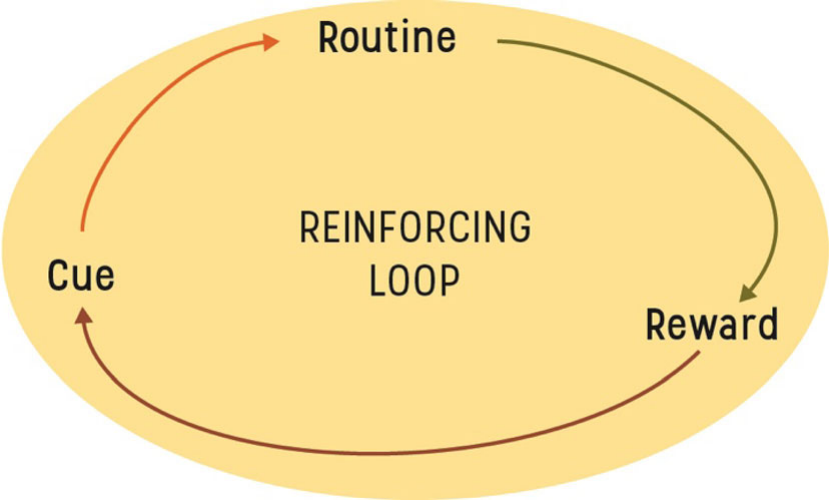
The habit loop illustrating how a habit is gradually reinforcing a rewarding association between cue and action (modified from Duhigg 2012)

The creation of habits is a repetitive process that gradually strengthens an association between a specific context and a behavioural response. With time, the reward that follows an action will be activated not by the action itself but by contextual cues (Wood and Rünger 2016). This habit formation process is sometimes conceptualized by “the habit loop” (see Fig. 1).

The importance of repetition in habit creation was illustrated in a study by Lally et al. (2010) in which repetitions of simple health behaviours (e.g. walking after dinner) required from 18 to as many as 254 days in the same context to become habitual and be performed without thinking. Habits can also be clumped together and activated almost as one unit through a phenomenon called “habit scripts” (Verplanken et al. 1994; Orbell and Verplanken 2018). This can be seen as a habit domino sequence where one habit activates the next one (and might explain how you end up at work without really remembering how you got there). These scrips, or routines, can involve multiple actions and sequences. For example, a nightly ‘dental care’ routine can include tooth-brushing and flossing as two separate habitual patterns (Judah et al. 2013). Although situations never completely map onto earlier experiences, research on transfer learning and stimulus generalization has shown that behavioural responses replicate quickly when the current environment is similar enough to the one in which the behaviour was previously performed (Bouton et al. 1999).

Context plays an important part in habit theory. Not only does the physical and social environment frame the range of possible actions and heavily steer habits creation, the context we repeatedly find ourselves in will with time contain numerous cues that activate behaviours we execute without much conscious oversight (Neal et al. 2012). Contextual cues are powerful drivers of behaviour and can include features of the physical environment, other individuals, emotions or preceding actions in a sequence (Wood and Rünger 2016). People can easily fall back on doing unwanted behaviours in a context with established contextual cues that they would not do in a context without the cues. Neal et al. (2011) highlighted this phenomenon in an ingenious experiment where they showed how some participants would eat stale, 1-week-old, popcorn at the cinema, but other participants would not eat similarly old popcorn in a meeting room, presumably because contextual cues for popcorn-eating was present in the former scenario but not the latter. Because people’s habits mainly operate by mechanisms they are not aware of, they tend to “own” their habits, and to describe them as intentional, particularly, if the habits are positive (Verplanken and Orbell 2003; Mazar and Wood 2018; Verplanken and Sui 2019). Research has shown that people often use post hoc justification for their habits. For example, we tend to make up reasons for unexplained automatic behaviour that align with our current values and goals (Adriaanse et al. 2018) and Neal et al. (2012) showed in an experiment that strong habits often were perceived as purposeful goal-directed behaviour, but were actually driven by recurring contextual triggers. Similar post hoc justifications for behaviour are also highlighted in cognitive dissonance theory which shows our tendency to justify actions when they are contradictory to our beliefs to reduce feelings of discomfort (Festinger 1962).

### Ways to break or create habits

There are several ways to break old or create new habits to be found in the literature. Some techniques help people adopt particular behaviours and allow people to act in intentional ways, while others assist the breaking of unwanted habits and help establish personal control over behaviour. We list some of the more prevalent ones below.

#### Implementation intention

Implementation intention is a strategy developed to bridge the intention–behaviour gap. It is an action plan approach to achieve pre-selected goal-directed actions. An implementation–intention strategy is used through committing to an if–then structure, i.e. “when situation X arises, I will perform response Y” (Gollwitzer and Sheeran 2006). The goal of the strategy is to have contextual cues activate an automatic behavioural response. If successful, the desired behaviour will occur without hesitation or deliberation in a pre-determined situation. Once the behaviour is set, through enough behavioural repetition, it may continue to operate as a new desired habit. Implementation intention can be used to promote new behaviours as well as breaking old habits, as it has proven successful in e.g. increasing consumption of organic food (Bamberg 2002) and recycling rates (Holland et al. 2006). Notably, implementation–intention strategies have shown an overall medium‐to‐large effect magnitude (*d* = 0.65) across 94 independent studies (Gollwitzer and Sheeran 2006).

#### Self-monitoring and cue identification

Because contextual cues automatically activate behaviour responses, careful self-monitoring and conscious inhibition of the action when it is activated in memory can be crucial in efforts to break free from unwanted habits. Quinn et al. (2010) showed that thinking phrases like “don’t do it” when an unwanted action response was about to occur was essential for controlling strong habits. Reflecting upon which situations habits occur in and identifying what internal and situational factors cue the habit through “cue-monitoring” have been shown to be effective in breaking unwanted habits such as unhealthy snacking (Verhoeven et al. 2014). Similarly, habit reversal training, a clinical behavioural treatment for harmful habits focuses on identifying the cue (specifically the sensation occurring just after the cue has been triggered) and replacing the cue–behaviour association with a competing behavioural response. The goal of the treatment is to alter the habit loop and link a more desirable behaviour to the already-established cue. This strategy has been successful in treating various damaging habits such as tic disorders and Tourette syndrome (Piacentini and Chang 2005; Fründt et al. 2017).

#### Habit discontinuity hypothesis

The habit discontinuity hypothesis poses that context change can offer an opportunity to break old and create new habits in situations where people free themselves from environmental cues that activate unwanted responses (Verplanken et al. 2008). Major discontinuities can be transitioning to new phases in life (e.g. from education to a job), geographical or physical changes (e.g. residential or work-related relocations), or changes in the environment where habits are executed (e.g. infrastructural changes). Initial empirical research supports this hypothesis and interventions to change seem to be more effective after relocation when habits momentarily “un-freeze” (Verplanken and Roy 2016).

## Three arguments for studying pro-environmental habits

It is made abundantly clear within the habit literature that our past behaviour is a strong predictor of future behaviour (e.g. Ouellette and Wood 1998; Sutton and Sheeran 2003). From a sustainability perspective, this does not bode well since many of our past behaviours support the unsustainable trajectory we currently find ourselves on. To ensure a sustainable future, we need to better understand the pull of our past decisions and explore the role of habits within research on sustainability transformations. This means both understanding how to break out of environmentally damaging behaviour patterns and how to establish new PEH. Below we present three arguments, showcasing the importance of looking at sustainable behaviours through a habit lens.

### Habit theory highlights how behaviour is heavily reliant on automatic processes

Habit theory highlights how our behaviour is heavily reliant on automatic processes, something often left out in sustainability narratives and in policy advice, as well as in pro-environmental research. A PEH approach would showcase how behaviour is heavily influenced by both deliberate and impulsive processes and how these interact. A habit perspective could situate sustainable actions as a part of a larger set of automatic actions and thoughts, pushing research and interventions beyond focussing on intrinsic motivation and reasoned behaviour alone.

Sustainability interventions that utilize our tendency to depend on the impulsive, automatic system can be effective tools for promoting certain sustainable behaviours. This perspective is exemplified in the behavioural economics literature that focuses on ‘nudges’. This research explores how automatic processes influence and guide behaviour more broadly e.g. how heuristics (cognitive rules of thumb), norms, information framing, loss aversion, and social pressure influence our choices and proposes interventions in alignment with automatic responses to encourage certain behaviours (Thaler and Sunstein 2008). Nudges have been successfully applied for triggering some shifts towards pro-environmental behaviours, for example, reducing water and paper consumption (Egebark and Ekström 2016), increasing food waste recycling (Linder et al. 2018), lowering energy use (Allcott and Mullainathan 2010; Allcott 2011; Costa and Kahn 2013; Allcott and Rogers 2014), and limiting food waste (Kallbekken and Sælen 2013). Similarly, a meta-analysis found that norms queued by the environment often result in automatic behaviour response and that such implicitly cued norms had a stronger impact on behaviour than explicitly stated norms (Bergquist et al. 2019). This was argued to be because implicit norms were less likely to result in anti-conformity responses such as psychological reactance when the behavioural response happens without conscious processing. Although nudges and norms are distinctly different from habits, these findings highlight the potential of interventions targeting automatic processes to promote pro-environmental behaviour changes. However, as nudge interventions are not always easy to implement (Ridder et al. 2020), research on PEH could move beyond ‘simple nudge approaches’ to a more holistic understanding of behaviour change. For example, a better understanding of habit theory can help tailor interventions so they are suitable for targeting different behaviours (Verplanken and Wood 2006). Less established or new behaviours, not bound by automatic processes, might align more easily with intentions and could therefore be targeted by more conventional attitude/intention building approaches (Dahlstrand and Biel 1997; Klöckner 2013). Routine behaviours that are guided by automatic processes might, however, demand interventions that account for habit breaking in order to change. PEH can guide the designing of interventions for pro-environmental behaviours by utilizing the already existing tools for breaking and creating habits (see the examples given in Sect. 2.5). On the whole, an important realization in the quest for sustainable change is that stronger interventions that account for habit breaking might be an essential, and currently underutilized, addition in order to address the automatic aspect of behaviour when aiming for urgently needed behaviour changes on the societal scale level.

### Environmental context sets boundary conditions for behaviour and shapes habits

Even though the environmental context is emphasized in habit theory, it is often overlooked in the literature on pro-environmental behaviour (Sörqvist 2016). This is a major shortcoming. Physical and social environmental conditions motivate and constrain actions through the range of behaviours they allow and enable. In order for any habit to develop, the possibility for that habit needs to be provided by the surrounding context. In ecological psychology, this range of possible behaviours is conceptualized as relations between features of the environment and abilities of the agent, also known as ‘affordances’ (Chemero 2003; Withagen et al. 2012). Sustainable behaviours have to be understood as part of the range of possible behaviours that the environment enables, for environmental attitudes and behaviour to align (Kaaronen 2017). And if a pro-environmental behaviour is made to be the easiest option, behaviour is likely to follow—regardless of values or intentions (Linder et al. 2021).

The habit literature highlights our tendency to develop habits when performing reoccurring actions in stable contexts (e.g. Gardner 2015; Wood & Rünger 2016), and that behaviour is heavily driven by context-specific cues after habits have been set. Together, this is making the environmental context a powerful leverage point for understanding and encouraging PEH. Considering that intrinsic motivation tends to fluctuate over time, a stable environmental context that supports reoccurring pro-environmental actions could be essential for the development of PEH. Furthermore, focussing on the behaviours we repeat in recurring contexts could increase understanding of inertia towards sustainable behaviour change, as habit theory highlights: it can be difficult to change behaviour in familiar contexts. Hence, reaching sustainability goals can be an uphill battle in conflict with everyday environments that automatically activate old unsustainable actions. This is exemplified by the above-mentioned habit discontinuity hypothesis, and mounting evidence supports this hypothesis in relation to pro-environmental behaviour. For example, university employees concerned about the environment who had recently moved house were commuting more sustainably than those who were equally concerned but had not relocated (Verplanken et al. 2008) and interventions to promote sustainable behaviours were more effective among newly moved participants (Verplanken and Roy 2016), suggesting that a change of context can provide an opportunity to more easily act on environmental values. Looking at sustainable behaviours through a habit lens would automatically link behaviour to the physical environment which might be needed to build an understanding of how sustainable behaviours are formed and steered by their surrounding context.

### Our habits and past behaviour shape our values and self-identity

**Fig. 2:**
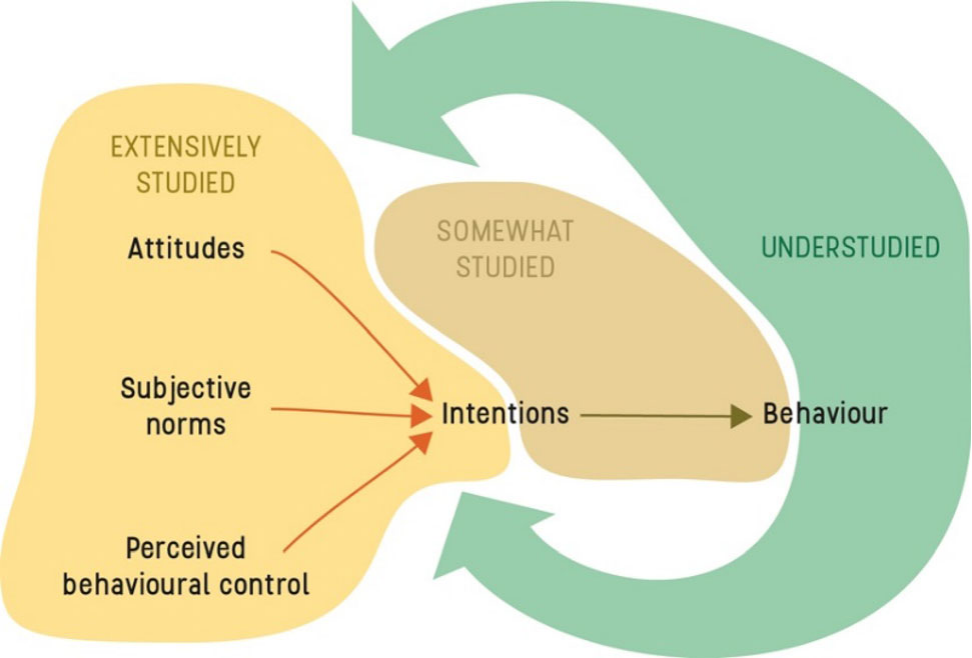
Visualizing the perceived research gap using the theory of planned behaviour as an example. Showcasing how PEH can complement the dominating models and narrative

A PEH research agenda would not only acknowledge that our past behaviour is a strong predictor of future action, but also that our habits could influence how we perceive ourselves, our values, beliefs, and self-identity. Recent studies have explored habits’ role in our identity and found that they may serve to define who we are (Verplanken and Orbell 2003; Verplanken and Sui 2019). By looking at their frequent behaviours people may label themselves as “the type of person that does X” (e.g. recycle, or buy organic food; Gardner et al. 2012) and infer that it is an important part of their identity. This aligns with self-perception theory that argues that we sometimes determine our attitudes and preferences by interpreting our actions (Bem 1972). Hence, a PEH lens on behaviour could contrast the assumptions made in the somewhat linear attitude–behaviour models by focussing on the feedback loop from behaviour back to attitudes (see Fig. 2). This amounts to conceptually flipping the models, highlighting how past behaviour and habits influence e.g. intentions and intrinsic motivation, and how habits, intention, and motivation co-evolve. PEH have been shown to correlate with biospheric values and norms (e.g. Verplanken and Roy 2016), although more research is needed to untangle the directions of causal relationships. Related research has shown some support for the importance of the feedback loop from behaviour to attitude. For example, Giusti et al. (2014) explored how physical access to nature experiences ensured a reoccurring interaction with nature that was shown to correlate with environmental awareness and sensitivity. Nature routines are an essential component to develop a meaningful relationship with nature (Giusti et al. 2018), which is true for intentional as much as incidental nature experiences (Beery et al. 2017). It has also been shown how personal experience with climate change increases intentions to act pro-environmentally (Broomell et al. 2015). Furthermore, a longitudinal study with 10-year-old children participating in a nature conservation project indicated that actively protecting endangered species can shape children’s connectedness with nature (Barthel et al. 2018). The above findings are not necessarily related to the developments of habits (although they certainly could be), other factors such as socialization (Klöckner and Matthies 2012) and social influence (Cialdini and Goldstein 2004) could be important factors in explaining how such activities influence our identity. But they showcase how our past behaviour and routines could be an important part of the development of sustainable values and identities. We believe that a PEH approach could be an important component to further explore this feedback loop from past behaviour, experiences, and habit to sustainable attitudes, values, and self-identity.

## Future outlook: Exploring habits’ role in sustainability transformations

Below we highlight some particularly promising research avenues and ways of applying PEH to advance the science on sustainable transformations based on the arguments presented above.

### Habit architecture—design urban environments to transform habits

Context is the invisible force that help creat and mentain habits. The immediate social and physical environment surrounding the individual sets boundaries for behaviours and can overpower people’s ability to act in line with pro-environmental values. As we develop habits by performing reoccurring actions in stable contexts, the behaviours these ‘stable contexts’ support or discourage are likely to heavily steer the creation of habits. Hence, careful design of everyday environments could greatly influence what kind of habits people will develop. Habit architecture (coined by Orbell and Verplanken 2018), i.e. promoting desired habits through careful design, seems especially important when considering the construction of projected new urban environments for roughly 2.5 billion people globally between 2010 and 2050 (United Nations 2014). Planning and carefully designing these new human habitats to promote PEH strikes us as a pragmatic leverage point in efforts to foster large-scale sustainability transformations. The habit discontinuity hypothesis (Verplanken et al. 2008) further highlights how these new constructions and urban landscapes might be seized upon as the basis of cost-effective opportunities to create PEH in new environments where people are more inclined to change (Verplanken and Roy 2016). If successful, PEH could become part of urban lifestyles that go in line with global sustainability goals. For example, habit architecture could be especially suitable for promoting transformations in mobility and commuting behaviour (Kaaronen and Strelkovskii 2020) and consumption habits (Wiedmann et al. 2020). These behaviours provide an intuitive example of applying a habit architecture since commuting travel (e.g. travelling to work) and everyday consumption (e.g. shopping at the local grocery store) is performed regularly in stable contexts and therefore likely lead to the development of habits. It has been shown that the extent to which daily transportation is carried out by walking or biking varies from less than 10% to more than 50% between cities of OECD countries (Buehler et al. 2017). A meta-analysis of 23 studies found the simple existence of sidewalks or footpaths to be the one factor most tightly coupled with walking behaviour (Wang et al. 2016). Suffice to say, pro-environmental attitudes only matter for travel behaviour insofar as the environment permits acting on them (Árnadóttir et al. 2019). Of course, the enabling of these pro-environmental behaviours does not guarantee habit formation. But it is safe to say that a context that enables and promotes sustainable actions would provide a good foundation for the development of PEH. An illustrative example is Copenhagen, a city that has witnessed a biking revolution in recent decades that is largely attributed to an increase in affordances for cycling, like cycling tracks, bicycle parking opportunities, bridges, and public bicycle schemes in parallel with policy for a cycling-friendly urban environment (Kaaronen and Strelkovskii 2020). In the 1960s, car use rapidly surpassed the use of bicycles in Copenhagen. However, a renaissance of the bicycle grew throughout the 1980s and 1990s. Recently, cycling represented 50% of all transport in the city while numbers of seriously injured or killed cyclists were decreasing. The increase in biking affordances in Copenhagen enabled pro-environmental cycling behaviour, whereby people developed stronger cycling habits, which in turn created a demand for the construction of more pro-environmental affordances for cycling. Such reinforcing feedback dynamics can lead to swift collective changes in behaviour once a critical threshold is passed (Kaaronen and Strelkovskii 2020). Thus, this indicates that tipping points in collective PEH formation could be efficiently triggered by changes in the physical urban form. Walking and biking are also promoted by making environments harder to drive in. Five cities in Germany, Austria, and Switzerland have witnessed reduced car share of trips through mutually reinforcing interventions like car-free pedestrian zones, shared streets with lower speed limits or wider sidewalks (Buehler et al. 2017). Crucially, behavioural change in all these cities has followed mainly from interventions in the physical environment, highlighting how habit architecture could complement interventions that appeal to people’s intentions or motivations to promote recurring sustanible actions.

### Pro-environmental habits to foster intrinsic motivation and sustainable cultures

A PEH approach might not only be an underutilized way to spur behaviour change but could also be an effective way to promote pro-environmental attitudes, identities, and cultures. An illustrative example of how a change in habits influenced norms and attitudes could be seen after the smoking ban in bars that happened in England in 2007. Not only did it reduced smoking behaviour overall but also significantly increased anti-smoking norms and increased the perceived risks of smoking (Orbell et al. 2009). Ultimately, we find sufficient evidence to hypothesize that sucesfully promoting PEH and nature routines could trigger self-reinforcing feedbacks that promote both people’s wellbeing (Giusti and Samuelsson 2020) and the psychological foundation of sustianable cultures (Giusti et al. 2014; Giusti 2019). Social–ecological systems research could address the issue of how to promote a co-evolution between PEH and environmentally conscious identities, especially among urban residents that are often psychologically disconnected from the Biosphere (Colding et al. 2020; Giusti et al. 2020). For instance, the research on urban stewardship may find novel empirical avenues on how habits and routines around actively caring for local ecosystems and species may co-evolve with intrinsic motivation (Andersson et al. 2017; Sanecka et al. 2020).

Another avenue that could be attractive for future research is to further explore how interventions that are already commonly used to break unhealthy habits (e.g. tobacco smoking, unhealthy diets, gambling) could be used for breaking recurring environmentally degrading behaviours. Lastly, when designing for PEH, sustainable behaviours need to be part of the range of possible behaviours the environment enables (Kaaronen 2017) and preferably be the easier ones to perform (Rosenthal and Linder 2021) and we see the need for novel research on how to ensure that pro-environmental motivations and attitudes are not overridden by repressive environmental features, preventing new PEH to form. Longitudinal studies following up on habit architecture and interventions, to explore when and if PEH develops could prove essential for building understanding on how to promote widespread sustainable habits that last over time—without effort and deliberation.

## Footnotes

1 Search phrase used: “sustainab* AND Habit”.

2 “Pro-environmental behavi* OR Proenvironmental behavi* AND Habit”.
